# A Participatory Interior Design Approach for a Restorative Work Environment: A Research-Intervention

**DOI:** 10.3389/fpsyg.2021.718446

**Published:** 2021-09-17

**Authors:** Margherita Pasini, Margherita Brondino, Rita Trombin, Zeno Filippi

**Affiliations:** ^1^Department of Human Sciences, University of Verona, Verona, Italy; ^2^Independent Consultant, Verona, Italy; ^3^Amnesty International Italia, Human Resources Department, Rome, Italy

**Keywords:** biophilic design, participatory design, environmental psychology, restorative environments, workplace, organizational well-being

## Abstract

Exposure to environmental stressors has physical and psychological consequences. A demanding physical environment involves the allocation of additional attentional resources and an increase in psycho-physical stress. This study illustrates the process of a research-intervention aimed at designing a workplace, using a participatory design approach, and considering the beneficial effect of restorative environments in reducing stressful elements and improving well-being at work. Stressful situations occur daily, compromising proper functioning while causing the occurrence of physiological and/or psychological disorders. To be able to safeguard their psycho-physical well-being, people normally adopt coping strategies, i.e., remedies that allow them to cope and manage situations that generate stress. One of these strategies is the exposure to natural environments, which promotes recovery and sustains psycho-physical well-being. The restorative properties of natural environments have been scientifically proven. However, even built spaces can be thought of as restorative environments, in particular when certain conditions are granted. An applied science, known as biophilic design, provides useful indications from this perspective. This project involved 57 employees of the Italian site of an international non-governmental organization, in the transition from a site no longer adequate to a new site requiring renovation. In a first phase, a survey was conducted, to verify the perceived quality of the current workplace and to detect the unmet workers' needs, and to assess some other important psychological constructs connected with perception of restorativeness and well-being. In a second phase, the findings emerged from the survey was analyzed in depth through a participatory interior design process, together with an interdisciplinary team of architects, technicians of the organization and environmental psychology researchers. The team, together with some representatives of employees, worked together through possible scenarios, adopting a biophilic design approach, to design the new workplace. At the end, the same survey of the first phase was conducted, to detect differences in perceived quality in the new workplace compared to the previous one.

## Introduction

### Workplace in an Historical Perspective

The nature of the workplace in the modern world has transformed drastically after World War II. In the 1950s, office layouts were inspired by the factory floor with rows-and-rows of desks cramming clerks tightly together. In parallel, executives enjoyed privileged amenities, such as private corner offices fitted with large windows through which they could make sure employees were hard at work. In the 1960s, as drinking and smoking were common in the office, the “three-martini lunch” was born. In 1978, ING Bank directors shared a vision for a new 49,982 m^2^ headquarters in Amsterdam. The focus of the building design was to maximize natural lighting, integrate organic art and install water features to enhance worker productivity and create a new image for the bank. After the relocation, absenteeism decreased by 15% and employees reportedly looked forward to coming to work and voluntarily tended to the planted vegetation features in the building (Romm and Browning, 1994). In addition to saving an estimated £1.3 million per year in operation costs from their new energy system and daylighting strategies, the new headquarters enhanced ING's image as a progressive and creative bank (Romm and Browning, 1994). In the 1980s people began to talk about and lobby for work-life balance, and wellness programs became a part of office life. Corporate culture was a priority. It was essential that businesses had a culture, defined it, and importantly that every employee knew about it in their cubicle fields. Whatever luster the cubicle had back in the 1960s, it faded away by the new millennium. The early 2000s saw the rise of open-floor office plans and telecommuting began. On the one hand, open-plan offices often lead to loss of productivity, problems with noise, temperature, and fatigue, increase of sickness, decrease of overall well-being of employees; on the other hand, open-plan offices save costs on real estate, increase communication and improve teamwork (Seddigh et al., 2014; Danielsson et al., 2015). Nowadays, companies aim for their employees to be fully engaged and productive, although rarely workplaces support their outcomes (Browning and Ryan, 2020). Lowered productivity can stem from noise distraction, attention fatigue, lack of sleep and bad moods (Maas, 2011). The physical workplace can either amplify or dampen many of the underlying factors of productivity. Hence, research in this sector is very valuable to touch on perspectives, challenges and lessons learned by project teams or owners when improving the workplace.

### Workplace and Its Effects on Stress

Stress management programs in the workplace typically focus on psychosocial factors and tend to disregard the growing body of research on workplace and environmental psychology (Van der Klink et al., 2001; Korpela et al., 2017a). Environmental psychology can be described as the study of the impact of the physical environment on people and the impact of people on the physical environment (Proshansky, 1987). It is an area of applied psychology, although a substantial portion of the research work is devoted to theoretical and methodological development, with only limited attention still to evidence-based results in this field. Common environmental psychology research settings are hospital, neighborhood, home, school and work environments (e.g. Andrade et al., 2013, 2017; Maxwell, 2016; Haapakangas et al., 2019; Mueller et al., 2019; Tobia et al., 2020). The bulk of environmental psychology research focuses on stress and stress-reducing effects of physical environments. Indeed, exposure to environmental stressors has physical and psychological consequences. Evidence shows that the office design influences employees' stress-level, productivity and well-being (Veitch et al., 2007; Vischer, 2007; Rashid and Zimring, 2008; Varjo et al., 2015; Kang et al., 2017). A demanding physical environment involves the allocation of additional attentional resources and an increase in psycho-physical stress. Exposure to environmental stressors appears to erode individuals' resilience, or ability to cope with additional task demands, reducing not only work performance but also overall well-being (Lamb and Kwok, 2016). As Vischer (2007, p. 178) claims: stressors in the work environment affect employee performance adversely when they are high intensity or prolonged, they slow down the individual's ability to process and understand the number and predictability of “signals,” which increase with task complexity. These sorts of stressors can be functional or symbolic (based on nonverbal communication of implicit intra-group meanings). Inadequate control over the physical environment is in itself a stressor that interferes with work objectives. Physical environments are perceived as more pleasant when people have some control on them, which contributes to a higher quality of work.

The design of a workplace also affects the quality of the work done by the people in it. Some of these effects are more direct: such as, visual or auditory stimuli which negatively affect concentration and consequently performance and are considered often problematic by employees (e.g., Haynes, 2008; Szalma and Hancock, 2011). More generally, all the functionally uncomfortable features of an office directly causes stress and reduces quality of work life (Vischer and Wifi, 2017). Others are more indirect, considering that, as people attach symbolic meanings to components of their workplace, this affects the way tasks are carried out. Workplace design influences worker performance, as it shapes mood, which in turn, influences how broadly or narrowly people think. In a synthesis of related research, Veitch (2012) points out that working under preferred conditions can create a state of positive affect (mood) that in turn leads to benefits in the form of increased cooperation, reduced competition, improved intellectual performance, and increased creativity. Schwartz and Porath (2014) surveyed 19,000 employed people to learn about the extent to which the thought of those individuals increased their work-related satisfaction and performance. The researchers found that people are quite aware of how their workplaces influence their performance: they normally feel and perform better and more sustainably when basic needs are met at four levels, such as renewal (physical), value (emotional), focus (mental) and purpose (spiritual). E.g., “the opportunity and encouragement to intermittently rest and renew our energy during the work day serves as an antidote to the increasing overload so many of us feel;” “feeling valued creates a deeper level of trust and security at work, which frees us to spend less energy seeking and defending our value, and more energy creating it;” “better focus makes it possible to get more work done, in less time, at a higher level of quality;” “the sense that what we do matters and serves something larger than our immediate self-interest is a uniquely powerful source of motivation” (Schwartz and Porath, 2014). Meeting even one of the needs in any of four core realms had a dramatic impact on every performance variable studied. Workplace design can contribute directly to feeling refreshed and valued and being able to focus on the task at hand. Schwartz and Porath's (2014) results are consistent with Leaman's (2003) findings: the better the occupants think the indoor environment is, the more likely people are to say they are productive, healthy, and happy (Leaman, 2003). Designing workplaces where people perform to the best of their ability is clearly a complex process. Research, however, can effectively guide designers' efforts in this direction. The physical-spatial dimensions commonly studied and analyzed concerning organizational outcomes are particularly: privacy, noise, natural and artificial lighting, air quality (Veitch, 2012).

### Restorative Environments and the Implication on Workplace Design

The concept of a restorative environment is linked to the notion of environmental stress. Stress occurs when environmental demands on people exceed their capacity to respond. Stressful situations emerge on a daily basis, compromising proper functioning, while also causing the occurrence of physiological and/or psychological disorders (Baroni and Berto, 2013). To be able to safeguard their psycho-physical well-being, people learn coping strategies, i.e., remedies that allow them to cope and manage situations that generate stress. One of these strategies is the exposure to natural environments, promoting recovery and sustains psycho-physical well-being. Existing theoretical frameworks, such as the biophilia hypothesis (Wilson, 1984), the attention restoration theory (Kaplan and Kaplan, 1989; Kaplan, 1995) and the stress restoration theory (Ulrich, 1983; Ulrich et al., 1991), suggest that nature contact can influence both productivity and well-being. The restorative properties of natural environments have been proven scientifically (Menardo et al., 2019). According to the literature (e.g., Kaplan, 1995; Korpela and Hartig, 1996; Pasini et al., 2014), some features of an environment enhance the quality of restoration in individuals. Kaplan (1995) identified four factors which characterize restorative environments: fascination, which refers to how an environment might attract the involuntary attention of a person; being away, which refers to how an environment causes a person to fell freed from everyday demands and obligations; extent, a characteristics which has two components, that are coherence which refers to how an environment is perceived as organized or not and scope that refers to how an environment offers the possibility of exploration; compatibility which refers to the correspondence between the characteristics of an environment and expectations of a person.

Restorative effects might appear after even very brief nature contact through so called micro-restorative experiences, particularly when stress levels are constant but not too high (Kaplan, 2001). Micro-restorative experiences include simple actions like glancing at a green landscape out of a window, or at a nature image on the wall, at an indoor plant, or other such similar experiences. For example, images of nature and nature view from the window reduce stress and anger, and help workers feel happier and healthier (Bringslimark et al., 2009; Korpela et al., 2017a). In the workplace, green plants can induce parasympathetic activity and greater stabilization of the autonomic nervous system (Ikei et al., 2014). Exposure to nature at work is related with well-being also longitudinally (Korpela et al., 2017b). Nature is restorative because it is filled with intriguing stimuli, modestly grabs attention in a bottom-up fashion, allowing top–down directed-attention abilities a chance to replenish. Unlike natural environments, urban environments are filled with stimulation that captures attention dramatically and additionally requires directed attention (e.g., to avoid being hit by a car), making them less restorative (Berman et al., 2008).

### A Biophilic Design Approach for Workplaces

Not only natural settings but also built spaces can be thought of as restorative environments (Ulrich, 1983; Kaplan, 1995; Berto et al., 2015). This has been effectively explored by biophilic design, an applied science that focuses on designing spaces with a special attention to certain characteristics, such as the level of environmental stimulation, coherence, complexity, the opportunity for visual contact with natural elements and the presence of biomorphic forms and structures. Biophilic design has received increased interest in recent years and is also being hailed as a strategy for reducing workplace stress while, at the same time, restoring attention, enhancing performance and increasing overall well-being. Numerous studies have been carried out highlighting the relevance of biophilic design in the workplace (Lottrup et al., 2013; Browning et al., 2014; Mangone et al., 2017). Considering possible effects of this approach, “Biophilic design promotes positive interactions between people and nature that encourage an expanded sense of relationship and responsibility for the human and natural communities.” (Kellert and Calabrese, 2015, p. 7).

A holistic biophilic workplace design strategy recognizes well-being as the aggregate of all our senses visual, aural, gustatory, olfactory, tactile, temporal. Our experience of tranquility relies on the harmonization of sensory inputs (American Society of Interior Designers, 2018), which may differ among build space user groups. Emphasis on effective daylighting, thoughtful spatial configurations, a multisensory experience and, when possible, natural ventilation strategies, along with interior greenery and ample views to nature, tends to create dynamic and healthful workplace experiences (Browning and Ryan, 2020).

The effect of restorative environments applied to workplace on organizational outcomes is still the subject of current research. Exposure to real nature enables better focus, mental stamina and productivity (Browning and Ryan, 2020). A simple device like strategic workstation orientation emphasizing a view to nature can have itself economic value by enhancing worker's performance and, thus, long-term productivity and profits (Heschong, 2003; Loftness, 2008). View and daylight quality can significantly affect how employees behave where they work, eat and break (Elzeyadi, 2011), as well as how much time is spent working at the office and sleeping at home (Figueiro et al., 2008; Boubekri et al., 2014). Noise-induced distraction has significant quantifiable negative impacts on ideation, reading comprehension, logical reasoning and useful interpretation of long-term memories (Banbury and Berry, 1998, 2005; Hongisto et al., 2008; DeLoach et al., 2015; Haapakangas et al., 2019). Attaching positive subjective meaning to the aural workplace experience can help combat noise distraction and associated health impacts. Nature-inspired acoustic treatments and water soundscapes can be incorporated to improve task performance and positive employee perception of well-being (Pheasant et al., 2010). Thus, financial repercussions of biophilic design in the workplace can be broken down into three categories: reduced absenteeism, improved cognitive performance and improved employee retention rate (from higher satisfaction). Each of these either saves a company money or increases their profit margin. A selection of research studies is summarized here: those with views of nature and daylight have 57 vs. 68 h of sick leave (Elzeyadi, 2011); views of nature are connected to 6–7% faster call handling, 8–16% improved cognitive performance (Heschong, 2003); indoor plants make to 10% improved task performance (CBRE, 2017) and 15% improvement (quicker and more accurate) in productivity tests (Nieuwenhuis et al., 2014); plants and window views are positively correlated with job satisfaction and organizational commitment (An et al., 2016); interior wood is related to an average. 10.7% reduction in completion time and 7.8% improvement in accuracy across 5 cognitive tests (Shen et al., 2020); nature sounds to an average of 13.9% increase in direct attention task score from before and after nature sound being used (Van Hedger et al., 2019); a combination of biophilic elements is connected to 14% improvement in short-term memory tasks (Yin et al., 2018). Biophilic changes made to a workplace can reduce absenteeism over a long term, limit complaints that drain human resource productivity and help retain employees over many years. Browning et al. (2012) calculated that the lost productivity value of absent employees engaged in office buildings in New York City is about $4.7 billion. Studies have shown that biophilic work environments can reduce about 10% of workers' absenteeism (Elzeyadi, 2011). Therefore, biophilic work environments could help a city such as New York City recoup $470 million in reduced absenteeism (Browning et al., 2012).

Bolten and Barbiero (2020) tried to identify the features that scientifically relevant publications describe (Browning et al., 2014; Sturgeon, 2017; Kellert, 2018) share together, so that they can be considered as essential ones. Bolten and Barbiero (2020) group them in the following categories: light, protection and control, air, views, greenery, curiosity, and materials, finishing and colors. The first group of three elements, standing on the authors comment, concerns the search for refuge, while the second group concerns search for resources, in an evolutionary perspective. They also noted that the acoustic aspect, namely “quiet and silence,” is never considered among these relevant publications, even if it is an important aspect (Berto and Barbiero, 2014). Exposure to a noisy environment for long periods can interfere with cognitive performance and affect physical and mental health (e.g., causing fatigue, irritability) which will influence workers' productivity indirectly (Banbury and Berry, 2005; Mak and Lui, 2012; Rasheed et al., 2019).

### Participatory Design: Design as Democracy

Participatory design is an approach attempting to actively involve multiple stakeholders (e.g., employees, partners, customers, citizens, end users) in the design process to meet their needs, while empowering them to be active shapers of their world. Participatory design is not a design style, rather a new perspective to processes and procedures of design. Recent research suggests that designers create more innovative concepts and ideas when working within a co-design environment with others as compared to when they generate ideas on their own (Mitchell et al., 2015; Trischler et al., 2018). The book Design as Democracy (Peña et al., 2017) defines Participatory Design as “hands-on democracy in action. It is grounded in the everyday places and lives of people. For over half a century it has guided us in understanding communities, honoring differences, creating vibrant neighborhoods and ecosystems, challenging environmental injustice, and fostering citizenship. Yet, in spite of our creative potential as designers, we tend to draw upon the same palette of techniques that were developed 50 years ago, without adapting or innovating for the contexts we now encounter.” This approach activates a participatory process relying on techniques and tools aimed at developing spaces that recognize and respond to fundamental human needs and rights. Participatory design has many applications in developing the built environment, particularly in relation to community regeneration projects (Kuiper, 2007). Giving employees more control over the design of their workplace makes a positive contribution to their well-being, according to a research study by the Helen Hamlyn Center for Design at the Royal College of Art, London, with architectural firm Helen Hamlyn Centre for Design Gensler (2017). However, data on its effectiveness and ways in which it can be applied to workplace design are poor. An evidence gap persists in research and interventions that adopt this approach. To address such limitations, the team set out to carry out a research-intervention project in the field of Interior Participatory Design to test out this approach empirically.

### The Aim of the Study

The aim of the present study is to illustrate a research-intervention project concerning the design of a workplace following some guidelines coming from environmental psychology research and biophilic design. The project involved employees and managers in the transition from a site no longer adequate for the needs of the organization to a new site to be renovated. The design suggestions are aimed to reduce stressful elements and improve the restorative qualities of the workplace. The intervention followed the Participatory Design approach, involving employees, managers, architects, internal designers, technicians, and environmental psychologists. To evaluate the effectiveness of the process, a pre-intervention ad a post-intervention assessment was conducted on employees, considering their perceptions of the quality of some features of the workplace, as well as other psychological variables.

## Method

### Research Design

This study illustrates an intervention research aimed to verify the effect of a participatory design intervention on the perceived quality of the physical features of the workplace. To do this, participants have been involved in three phases: a first assessment (T1), to measure the perceived quality of some features of the workplace, a second phase (Intervention) consisting in the participatory design intervention, and a final phase (T2), aimed to assess the same variables measured in the first phase, to detect changes in satisfaction about the quality of the workplace. The study involved all the employees and the managers of the Italian site of an international non-profit organization during the transition period from the old site to a new site to be renovated. A small team involving one manager, an architect, and the environmental psychology researchers was created first, to discuss the research-intervention project. This project was presented in an assembly session to all the employees. In this assembly the staff was informed about the project and trained on the state of art of the research related to biophilic design and potential restorative characteristics of work environments and their impact on workers well-being.

In the same context, an on-line survey was launched to verify employees' satisfaction for the quality of some features of the current workplace, to detect the workers' unmet needs. The on-line survey was available for 2 weeks. Six months later, the organization moved to the new site, and 2 months later, the same survey was conducted to assess the change in quality satisfaction for the old site compared to the new one. The survey was available for 2 weeks here too. The surveys were conducted online, and all participants agreed to participate after filling in the informed consent.

After the first assessment wave, a participatory interior design process started, including volunteers among the employees, together with an interdisciplinary team of architects and technicians of the organization, supported by the environmental psychology researchers. The team worked together through possible scenarios adopting biophilic design, designing together the interiors of the new workplace.

The participatory design process consisted of two steps. The aim of the first step was to develop proposals for the interior design of the new headquarters. In this step employees were assigned to three groups based on their work unit and role. For each group, the work was developed in four sessions. In the first session, titled “*Creativity takes courage*,” participants were asked to individually draw “The office I would like” on a blank piece of paper. At the end of this session all the drawings were shared between all the participants. In the second session, titled “*None of us is as smart as all of us together*,” participants were asked—divided into small groups—to “draw the formal and informal common spaces of the new headquarters” using the plans of the new headquarters. In the third session, title “*Description of the drawings*,” all the drawings were shared by all the small groups in a plenary session. In the last session, titled “*Reflections and feedback*,” the proposals were discussed by participants and the interdisciplinary team of architects and internal technicians with the support and the facilitation of the researchers.

In the second step of the participatory design process, a small group composed by employees' representatives and the interdisciplinary team of architects and internal technicians, with the support of the environmental psychology researchers, analyzed the proposals developed in the first step. The aim was to identify the technical and economical sustainability of the proposals and to find a design solution, good enough for all the parties. The biophilic design perspective was strongly considered, to find the most suitable final solution, taking into account all the possible aspects, such as the restorative qualities of the environment, light, acoustic, air, as well as the presence of views of nature and the visual contact with natural elements, and the presence of biomorphic forms and structures.

At the end of this step, the identified design solutions were shared with all the employees in a plenary session.

### Participants

The participants were employees of the Italian site of an international non-profit organization who, on voluntary basis, decided to participate in the research-intervention project. 55 workers have been invited to participate at the first wave, and 57 workers at the second one (the same of the first invitation, and 2 more new workers). At the first wave of the survey 39 employees participated (response rate: 71%), belonging to 12 work units. 74% were females. Considering age, 16 (41%) were in the range 30–39, 16 (38.5%) were in the range 40–49, and 8 (21%) were over 49. Considering the educational level, 9 (23%) had a high school degree, 4 (10%) a bachelor's degree, 18 (46%) a master's degree, and 8 (20.5%) a PhD or a post graduate degree. They were mainly officers (77%, 23% heads of a unit) and most of them worked in the non-profit organization from more than 5 years (77%). The participatory design process involved 24 of them belonging from all the work units. At the second wave of the survey 51 employees participated (response rate: 89%). 715% were females. Participants' age was as follow: 3 (6%) were under 30, 18 (35%) were from 30 to 39, 20 (39%) from 40 to 49, and 10 (20%) over 49. Regard to the educational level, 11 (22%) had a high school degree, 8 (16%) a bachelor's degree, 21 (42%) a master's degree, and 11 (22%) a PhD or a post graduate degree. Again, participants were mainly officers (73%, 27% heads of a unit) and most of them worked in the non-profit organization for more than 5 years (78%). A total number of 61 participants were involved, and 26 of them participated in both surveys.

### Measures

Physical environment perception was assessed, as well as some measures of psychological well-being.

Physical environment perception was assessed considering two main measures. The first one was related to the *perceived quality of the physical features* of the workplace, following the conceptual framework developed by Bolten and Barbiero (2020). The second one was related with *perceived restorativeness* (Ulrich, 1983; Kaplan, 1995), applied to the work environment.

*Perceived quality of the physical features* was assessed with 20 *ad-hoc* items to evaluate workers' satisfaction for the workplace in five dimensions: natural light/light control, air, view connected with nature, acoustic comfort, and destress areas. For all these dimensions from 3 to 5 items has been formulated, and internal consistency was verified both for the first and the second assessment, using Cronbach's alpha. All responses were given on a 6-point Likert scale from 1 (not at all satisfied) to 6 (completely satisfied).

*Natural light/light control* (4 items) assessed the satisfaction for natural light, the possibility of controlling light in the personal work area, and the adequacy of the light in the personal work area. An example of item is: “Evaluate how much you are satisfied for the quality of your workplace, in respect of natural light.” Cronbach's alpha was 0.90 on T1 assessment and 0.91 on T2 assessment.

*Air* (4 items) assessed the quality of thermal comfort, control of thermal control, and smell. An example of item is: “Evaluate how much you are satisfied for the quality of your workplace, in respect of the thermal control.” Cronbach's alpha was 0.89 for T1 and 0.95 for T2.

*View connected with nature* (3 items) evaluates the satisfaction for real nature (plant, green, water, landscape), artificial nature (artificial plants, poster of nature), and the presence of objects recalling local ecological characteristics of the environment. An example of item is: “Evaluate how much you are satisfied for the quality of your workplace, in respect of the view on natural green (e.g., plants, water, greenery, landscape, …).” Cronbach's alpha was 0.91 for T1 and 0.90 for T2.

*Acoustic comfort* (4 items) concerned the evaluation of quiet and silence, and acoustical privacy. An example of item is: “Evaluate how much you are satisfied for the quality of your workplace, in respect of acoustic distractions (e.g., phone rings, conversations.” Cronbach's alpha was 0.85 for T1 and 0.95 for T2.

*Destress areas* (5 items) assessed the satisfaction for break areas, coffee break and lunch break areas, areas for distressing events and purposes. An example of item is: “Evaluate how much you are satisfied for the quality of your workplace, in respect of the lunch break area.” Cronbach's alpha was 0.80 for T1 and 0.91 for T2.

*Restorativeness of work environments* was assessed within an adaptation of Rest@work scale (Pasini et al., 2011) composed by 12 items related to three dimensions: physical and/or psychological “*being-away*” from demands on directed attention, four items, Cronbach's alpha 0.63 for T1 and 0.69 for T2; “*fascination*,” a type of attention assumed to be effortless and without capacity limitation, 3 items, Cronbach's alpha 0.80 for T1 and 0.86 for T2; the “*coherence*” perceived in a workplace, 5 items, Cronbach's alpha 0.69 for T1 and 0.60 for T2. Answers were given on a 7-point Likert scale, from 1 = strongly disagree to 7 = strongly agree. Examples of items are “My workplace awakens my curiosity” (Fascination), “My workplace is well organized, and I can easily find what I need” (Coherence), and “My workplace is designed as a place in which I can take some small breaks to think at pleasant things sometimes” (Being-Away). Cronbach's alpha for the whole scale was 0.72 for T1 and 0.77 for T2.

The following outcomes variable for psychological well-being were chosen:

*Physical and Psychological* well-being was assessed with the General Health Questionnaire (GHQ; Goldberg and Williams, 2000), a standardized scale which is often used to measure psychological functioning. The validity and reliability of the 12-item version has been extensively evaluated (e.g., Piccinelli et al., 1993; Balducci et al., 2017). Six items are focused on positive mood states conditions (e.g., ability to concentrate, feeling useful) and six are focused on negative mood states conditions (e.g., loss of sleep, inability to overcome difficulties). Respondents are asked to indicate how frequently (in the last 15 days) they have experienced the different symptoms, with a four-points rate scale (positively phrased labels: “more so than usual,” “same as usual,” “less than usual,” “much less than usual;” negatively phrased labels: “not at all,” “no more than usual,” “rather more than usual,” “much more than usual”). The Likert method (all items coded 0–1–2–3) has been used for the scoring, with a higher score indicating worse physical and psychological well-being. Cronbach's was 0.77 for T1 and 0.88 for T2.

*Work engagement* was assessed with the 3-item version of the Utrecht Work Engagement Scale (Schaufeli et al., 2019). Each item represented a dimension of work engagement: (1) “At my work, I feel bursting with energy” (vigor); (2) “I am enthusiastic about my job” (dedication); (3) “I am immersed in my work” (absorption). Responses were given on an 8-point Likert scale (1 = “never;” 8 = “always”), and Cronbach's was 0.85.

*Job satisfaction* is evaluated with a single-item measure. Respondents are asked to rate their overall job satisfaction on a scale from 1 (very unsatisfied) to 8 (very satisfied).

Unfortunately, work engagement and job satisfaction were measured only on the second wave survey, for organizational reasons. An organizational climate analysis had been carried out only few weeks before the beginning of this project, and it was decided it was not appropriate to repeat a similar analysis so close to the previous one, while for the second wave there was not this problem, and the two variables could be assessed.

## Results

### Participatory Interior Design

#### Step 1: Proposals' Development

**Figure 1:**
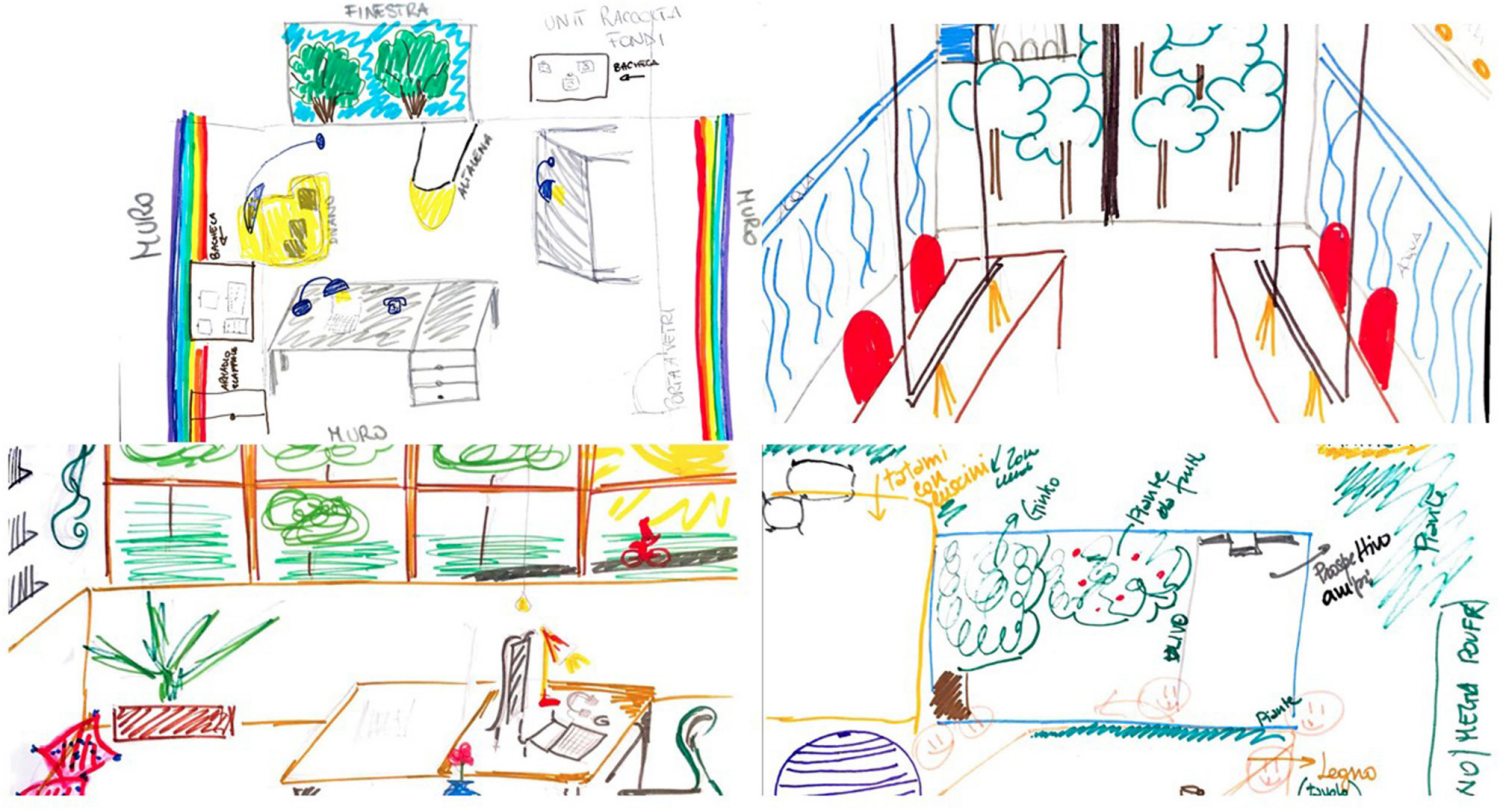
Examples of participants' drawings, for the session “The office I would like” (Step 1 - Session 1).

The main results of the first step of the participatory interior design process were the groups proposals for the interior design of the new headquarters developed after the four work sessions. Already in the first session “*Creativity take courage*,” in which participants were individually invited to draw the office they would like, the drawings showed characteristics related to the biophilic design (see Figure 1).

In fact, most of the drawings reported the opportunity for visual contact with natural elements and the presence of biomorphic forms and structures. In particular in the individual drawings, some elements recurrently emerged among all units (reported in order, by frequency and relevance): plants (on the floor, windows, desks, wall); large windows and natural light (views of nature and of the historical city where the building is located); large desks (L shape, rectangular, squared); joined desks; office and desk lights giving the effect of natural light; comfortable chairs, with wheels, ergonomic; use of wood for tables and furniture; extra seats in every room (stools with wheels, armchairs, sofas).

**Figure 2:**
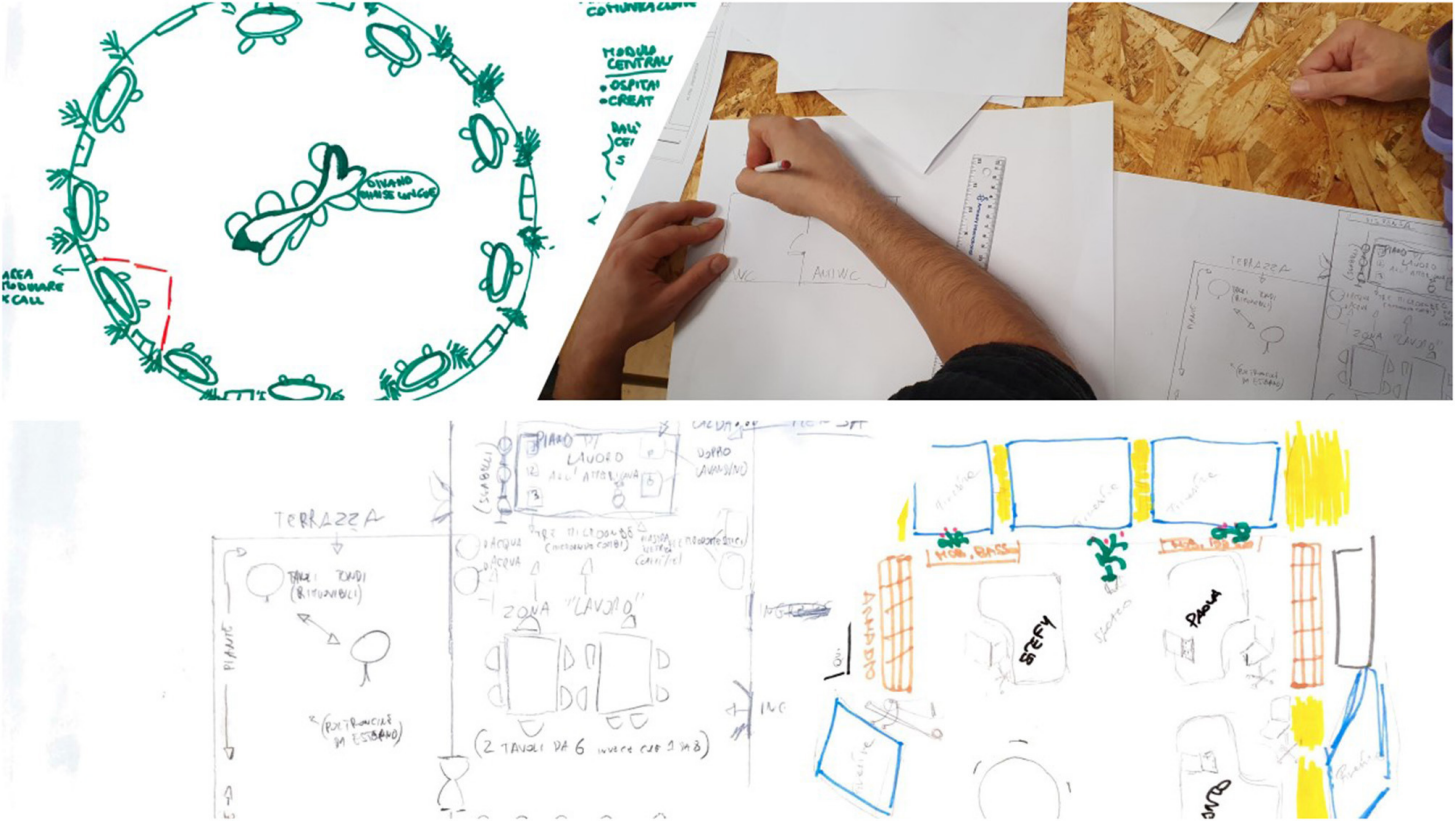
Examples of participants' drawings of the formal and informal common spaces of the new headquarters (Step 1 - Session 2).

In the second session, “*None of us is as smart as all of us together*,” when small groups worked on the design of the formal and informal common spaces using the plants of the new site, most of the participants underlined the need of features of places recalling natural environments (see Figure 2).

The groups elaborated their proposal for informal and formal common spaces beginning from their desires and then using the floorplans to choose the best solution for each space.

Considering informal spaces, silence/privacy area essentials were considered as very important by most of the participants. Suggested amenities included: a small light-refreshment area offering coffee, herbal teas, water, apples and seasonal fruit; warm and soft light; soft seating arrangements environment including poufs, hugging armchairs, leaning beds; again plants. They also proposed colored LEDs in the bathroom for relaxation. For the canteen they chose to connect the kitchen to the terrace to have the possibility to use the terrace for the lunch break and other breaks during the work time. Terrace essentials were considered as very important by the majority of the employees. For these places the groups proposed: a small vegetable garden or windows with aromatic plants was proposed; natural wood furniture getting rid of laminated items; elimination of plastic (e.g., coffee machines with biodegradable pods; personalized ceramic cups; separate garbage collection; biodegradable bags; absence of plastic utensils; minimal use of paper; natural foods in snack dispensers); a water dispenser (not inside a plastic tank).

Considering formal common spaces, for instance, reception hall essentials have been considered as very important by the majority of the participating group. General preference has been to get rid of chairs and armchairs and glass top tables in favor of organizing a sofa area, fitted with a soft/upholstered bench, or a long wooden seat with additional armchairs and a display containing magazines and promotional materials concerning the organization. Again, suggestions included plants, a refreshment corner with coffee and a water dispenser for visitors. Designing the conference room, the employees pointed out to the need to have potted plants; use natural materials, especially wood; set up a small stage/platform hosting organizational meetings and a wheelchair access ramp; a TV screen with running images; a projector positioned in the middle of the room; a raised table with the logo of the organization.

After the third and fourth sessions, aimed at sharing the small groups drawings in the plenary session and discussing the proposals with the interdisciplinary team of experts, the architect elaborated the first draft of the project.

#### Step 2: Identified Solution and their Implementation

In the second step the final project was drawn up by the architect based on the participants' proposals. A first draft was discussed with representative of the participants of the first step and then finalized after their agreement and that of all the employees.

**Figure 3:**
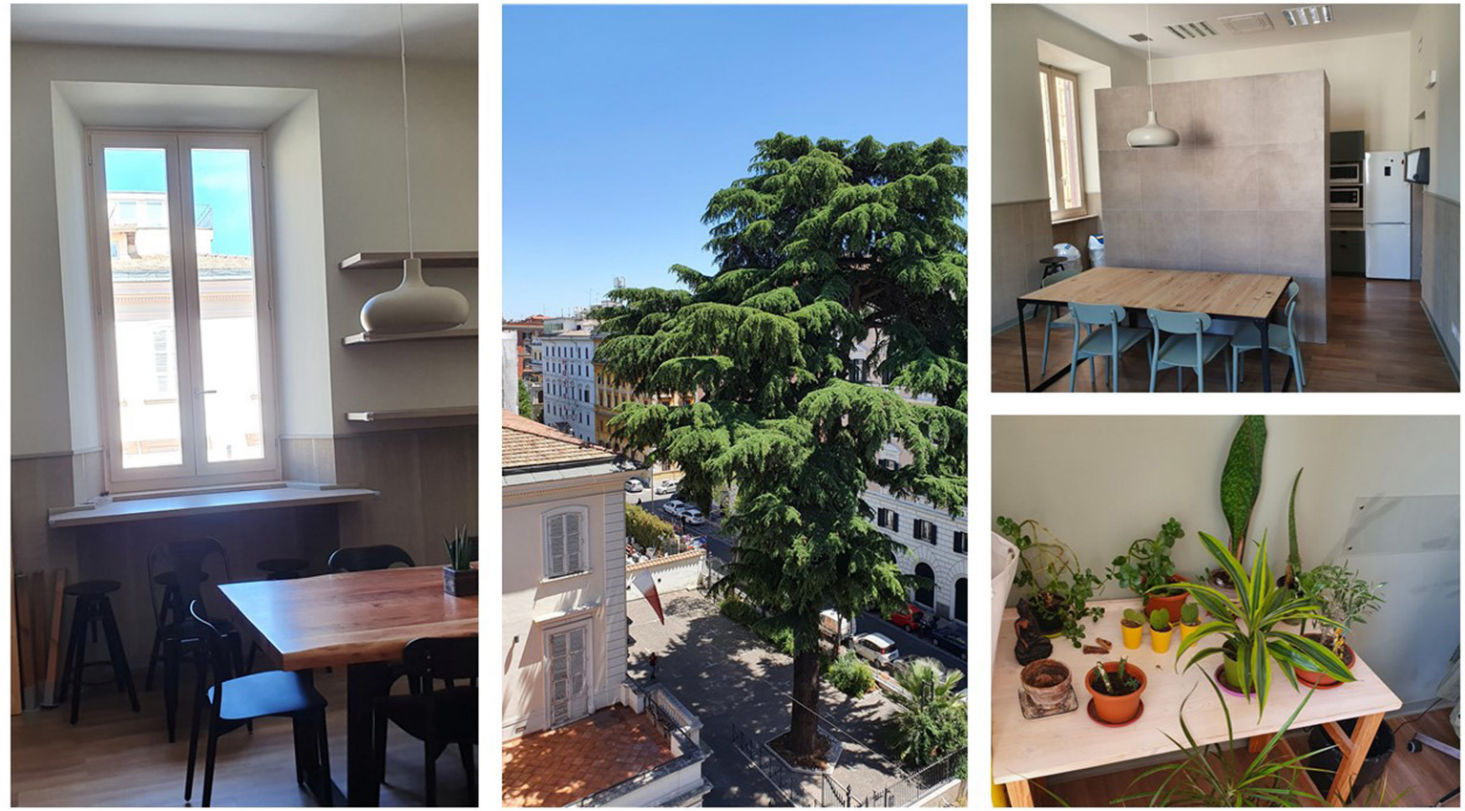
Some views of the new headquarters.

The following are the characteristics of the new headquarters which highlight the attention to the biophilic design (see Figure 3).

*Natural light/light control*—the new workplace is provided with very high ceilings and windows to make the most of sun exposure to both south, west, east and north sides. This allows to maximize natural sunlight, while minimizing electric light use.

*Air*—Operable windows and curtains give employees the opportunity to control both temperature and air variability in the office. Natural ventilation is therefore maximized, while artificial air is minimized.

*View connected with nature*—in order to maximize inside and outside views, two open spaces, namely a balcony and a terrace were created. They are dedicated to moments of restoration, evoking a sense of perspective and refuge.

*Destress areas*—small green spaces were customized by employees who also look after them (see photos) taking the advantage of micro-restorative experiences. Outdoor rest areas support recovery and engagement opportunities with nature (i.e., plants, water, animals). Here, employees meet and have conversations, while enjoying the sky and finding relief from the boundaries of the office space. A dining room was designed to evoke a feeling of being “at home” (see photo), while functioning as a central focal point that keeps people united, involved and connected.

*Connection to a shared mission and values*—transparent doors, posters and paintings of the historical milestones of the organization (see photo) were placed in the office to inspire employees, while conveying the organization's values.

*Natural materials and colors*—throughout the building laminate flooring connects to nature (see photo), furthermore each team had the opportunity to choose from a range of natural colors to personalize the walls of their own office.

### Survey Results

#### Descriptive Statistics and Improvement in Perceived Quality of the Workplace

The first part of Table 1 summarizes the main results concerning the descriptive statistics (mean, standard deviation and sample size) for the psychological variables considered in the study. We are going to describe them looking at the two moments of the assessment, the first referring to the old site, and the second referring to the new one, which has been design after the participatory design process, looking at the possible aspects, such as the restorative qualities of the environment, light, acoustic, air, as well as the presence of views of nature and the visual contact with natural elements, and the presence of biomorphic forms and structures.

**Table 1:** Descriptive statistics for the considered variables in the two surveys, and results of the paired sample *t*-test comparing first and second assessment on the same variables.

	**Scale range**	**N T1**	**Mean score T1 (SD)**	**N T2**	**Mean score T2 (SD)**	**Mean difference (T2–T1)**	***t***	***df***	***p* value**	**Cohen's d**
**Natural light/light control**	1–6	39	2.6 (1.3)	47	4.1 (1.2)	1.5	4.5	25	**<** **0.001**	**0.88**
**Air**	1–6	39	2.7 (1.3)	45	4.3 (1.0)	1.8	6.1	24	**<** **0.001**	**1.21**
**View connected with nature**	1–6	39	1.6 (0.9)	47	3.0 (1.3)	1.7	6.8	25	**<** **0.001**	**1.34**
**Acoustic comfort**	1–6	39	1.5 (0.6)	46	3.7 (1.2)	2.3	9.3	24	**<** **0.001**	**1.85**
**Destress areas**	1–6	39	2.1 (0.9)	47	3.5 (1.1)	1.4	5.3	25	**<** **0.001**	**1.03**
Being-away	1–7	39	3.5 (0.6)	47	3.4 (0.8)	−0.2	−1.2	24	0.259	
Fascination	1–7	39	2.7 (1.0)	47	3.0 (1.2)	0.5	1.6	24	0.115	
**Coherence**	1–7	39	2.8 (1.0)	46	4.2 (0.8)	1.2	4.9	24	**<** **0.001**	**0.99**
**Overall perceived restorativeness**	1–7	39	3.0 (0.6)	47	3.6 (0.7)	0.6	2.9	24	**0.008**	**0.58**
GHQ—Physical and Psychological Well-being (high scores means low mental health)	0–3	39	1.9 (0.4)	48	2.0 (0.4)	0.1	1.1	25	0.273	
Work engagement	1–8			48	5.3 (1.6)					
Job satisfaction	1–8			48	5.5 (1.3)					

*Significant improvements are highlighted in bold*.

A first set of variables concerns the Perceived quality of the physical features: natural light/light control, air, view connected with nature, acoustic comfort, destress areas. The level for the first assessment, which considered employees' perceived quality of these characteristics referring to the old site, is quite low, going from the highest value of the air quality (M = 2.7, SD = 1.3), considering a scale from 1 to 6, to the lowest value of acoustic comfort (M = 1.5, SD = 0.6). One of the most important aspect, considering the biophilic design perspective, that is view connected with nature, shows a very low level as well (M = 1.6, SD = 0.9).

Perceived restorativeness of the old site also shows low values, with no one of the dimensions reaching at least the medium level of 4 on the 1-to-7-point scale. The overall perceived restorativeness score is 3 (SD = 0.6), and the lowest score is for the dimension “fascination,” with a mean score of 2.7 (SD = 1.0).

Mental health, evaluated with the General Health Questionnaire, shows a quite good score (M = 1.9, SD = 0.4). A score higher than 2 should ought to alarm about the possibility of low mental health. This score was mainly attributed to their personal work condition (61%) and to both home and work condition (29%), while only 10% attributed their answers only to their private life. This means that this score, in this sample, can be considered a valid way to assess the quality of work condition for employees' well-being.

Looking at the same variable assessed in the second survey, when employees had to evaluate the new workplace, the majority or the variable showed an increased level of perceived qualities of the physical environment and restorativeness. Descriptive statistics were computed considering all the respondents at the second survey (48 employees), while a paired sample *t*-test were performed considering the 26 participants who gave their responses participated in both the surveys.

The scores about the perceived quality of the physical features ranged from the lowest value of view connected with nature (M = 3.0, SD = 1.3) to the higher score for air quality (M = 4.3, SD = 1.0), on a 1-to-6-point scale. Perceived restorativeness ranged from the lower score for the dimension “fascination” (M = 3.0, SD = 1.2) to the higher score of “coherence” (M = 4.2 SD = 0.8).

Comparing results of the two assessments, almost all the variables showed better scores in the second survey (the one considering the new site). Results of paired sample t-test are reported in Table 1, in the last five columns. Large effect size was found for the improvement of perceived quality of all the five aspects of physical environment, with Cohen's d ranging from 0.88 (natural light/light control) to 1.85 (acoustic comfort).

About perceived restorativeness, being-away and fascination did not significantly change, whereas coherence highly improved (Cohen's d = 0.99). Considering together the four dimensions, an improvement in overall perceived restoration was found, with a medium size effect (Cohen's d = 0.58).

No significant changes were found in mental health, even if the score decreased from 1.9 to 1.1, that is an indicator of better mental health. Even in the case of the second survey, the scores were mainly attributed to the work condition (60%) or both to work and private life (30%) and only 10% of respondents attributed their answers at the General Health Questionnaire to their private life. This allows us to consider mental health as strongly connected to work experience in our sample.

The second survey also assessed two additional psychological states which can be considered indicators of well-being at work: work engagement and job satisfaction. As shown by Table 1 (last two rows), the level of both variables is quite high, with a mean score of work engagement of 5.3 (SD = 1.6) and a mean score for job satisfaction of 5.5 (SD = 1.3) on a 1-to-8-point scale, which are on the top half of the scores. Nevertheless, not having a comparison with the data of T1, the analysis of these results does not give much information. The analysis of relationships between well-being indicators and the environmental quality evaluation could probably give more interesting information. This kind of analysis is described in the following paragraph.

#### Relationships Among Perceived Quality of the Environmental Features, Restorativeness and Well-Being

A second step of the analyses concerned the relationships among the variables considered in the T1 and T2 surveys. Table 2 show Pearson's correlation coefficients between the perceived quality of the five aspects of physical features in the workplace, separately for T1 assessment (below the diagonal) and T2 assessment (above the diagonal).

**Table 2:** Pearson's correlation coefficients between the study variables about quality and restorativeness of the workplace (T1 assessment below the diagonal, N1 = 39, and T2 assessment above the diagonal, N2 = 48).

	**1**	**2**	**3**	**4**	**5**	**6**	**7**	**8**	**9**
1. Natural light/light control	–	0.53^***^	0.54^***^	0.45^**^	0.47^***^	0.25	0.31^*^	0.32^*^	0.40^**^
2. Air	0.58^***^	–	0.63^***^	0.47^**^	0.64^***^	0.15	0.43^**^	0.29	0.39^**^
3. View connected with nature	0.60^***^	0.13	–	0.38^**^	0.59^***^	0.39^**^	0.44^**^	0.34^*^	0.56^***^
4. Acoustic comfort	0.37^*^	0.29	0.46^**^	–	0.46^**^	0.24	0.51^***^	0.27	0.45^**^
5. Destress areas	0.48^**^	0.52^***^	0.34^*^	0.56^***^	–	0.33^*^	0.65^***^	0.60^***^	0.73^***^
6. Being-away	0.17	0.45^**^	0.26	0.39^*^	0.39^*^	–	0.50^***^	0.16	0.66^***^
7. Fascination	0.32^*^	0.50^**^	−0.06	0.19	0.31	0.43^**^	–	0.20	0.74^***^
8. Coherence	0.20	0.16	0.13	0.40^*^	0.25	0.16	0.21	–	0.72^***^
9. Overall Perceived Restorativeness	0.32^*^	0.45^**^	0.15	0.47^**^	0.42^**^	0.61^***^	0.68^***^	0.80^***^	–

* *p < 0.05*,

** *p < 0.01*,

*** *p < 0.001*.

The correlations among the five aspects of the workplace that has been evaluated, that is natural light/light control, air, view connected with nature, acoustic comfort, and destress areas, are all significant in the T2 survey, and almost all significant in T1 survey, excepted for the relationship between air and view connected with nature, and air and acoustic comfort. Natural light/light control is related with view connected with nature in both the surveys, with a strong correlation coefficient, higher than 0.50 (*p* < 0.001 in all cases). Also, satisfaction for the destress areas are highly correlated with all the other aspects, mainly with air and acoustic comfort in the evaluation of the old site, and with air and view connected with nature in the evaluation of the new site.

An interesting result concerns the correlation between the perceived quality of the physical features, in the 5 aspects, and the perceived restorativeness of the work environment. Results on the old site show a correlation between the overall score of restorativeness and the perceived quality of 4 of the 5 workplace features. It's difficult to understand why no correlation was found between overall perceived restorativeness and view connected with nature. Nevertheless, it's possible to note that the variability in the first sample on these two variables is low, and this can lower the correlation between these two variables (Goodwin and Leech, 2006).

Looking at the results for the new site, correlation between perceived restorativeness and perceived quality of the physical feature in the new workplace are all significant, except for coherence and being-away. The higher correlation is between perceived quality of the destress areas and overall perceived restorativeness of the workplace (*r* = 0.73, *p* < 0.001). This means that when people perceive a good quality of destress areas, they also perceive the workplace as a restorative environment. The perceived quality of the destress areas seems also to be strongly connected with two dimensions of perceived restorativeness, that is fascination (*r* = 0.65, *p* < 0.001) and coherence (*r* = 0.60, *p* < 0.001). Finally, the perceived quality of only one aspect of the physical workplace correlates with all the dimension of perceived restorativeness, that is view connected with nature (being-away: *r* = 0.39, *p* < 0.01; fascination: *r* = 0.44, *p* < 0.01; and coherence: *r* = 0.34, *p* < 0.05).

Finally, we were interested in exploring the relationship between physical environment perceptions and psychological well-being outcomes. Table 3 shows Pearson's correlation coefficients between perceived quality of the physical features and restorativeness of the workplace, and psychological well-being outcomes. Considering results at T1, in which the only psychological well-being measure collected was mental well-being, measured with GHQ-12, a significant correlation was found between the dimension “coherence” of perceived restorativeness and GHQ-12 (r = −0.32, *p* < 0.05). Considering GHQ-12 scoring method, in which higher scores indicate worse mental health, the negative correlation means that high perceived coherence is associated with high mental well-being.

**Table 3:** Pearson's correlation coefficients between the study variables about quality and restorativeness of the workplace and psychological well-being outcomes.

**Physical environment perception^a^**	**Mental well-being^b^ (T1)**	**Mental well-being^b^ (T2)**	**Work engagement (T2)**	**Job satisfaction (T2)**
Natural light/light control	−0.03	−0.11	**0.34** ^*^	**0.50** ^***^
Air	0.02	−0.05	**0.34** ^*^	**0.34** ^*^
View connected with nature	0.04	**−0.30** ^*^	**0.48** ^***^	**0.54** ^***^
Acoustic comfort	−0.01	−0.17	0.14	0.19
Destress areas	0.07	−0.06	**0.54** ^***^	**0.58** ^***^
Being-away	−0.01	**−0.33** ^*^	**0.36** ^*^	0.18
Fascination	0.04	−0.13	**0.42** ^**^	**0.48** ^***^
Coherence	**−0.32** ^*^	−0.26	**0.37** ^*^	**0.36** ^*^
Overall perceived restorativeness	−0.20	−0.26	**0.47** ^***^	**0.47** ^***^

* *p < 0.05*,

** *p < 0.01*,

*** *p < 0.001*.

*Significant correlations are highlighted in bold*.

a *Correlations are computed between variables assessed at the same timepoint, T1 or T2*.

b *High score means low physical and psychological well-being*.

The analysis of correlations between well-being outcomes and perceived quality of the physical features in the new workplace and restorativeness dimensions highlighted the importance of the possibility of a view connected with nature in relation to all the outcomes. The strongest relationship was found with job satisfaction (*r* = 0.54, *p* < 0.001), followed by work engagement (*r* = 0.48, *p* < 0.001) and by physical and psychological well-being (*r* = −0.30, *p* = 0.044). Natural light and light control, and destress areas seemed more related with job satisfaction than with work engagement, respectively (job satisfaction: *r* = 0.50, *p* < 0.001; *r* = 0.58, *p* < 0.001; work engagement: *r* = 0.34, *p* < 0.001; *r* = 0.54, *p* < 0.001). For the quality of the air the relationship with both the outcome variables was.34 (*p* = 0.022). Considering restorativeness, we found only a positive correlation with work engagement and job satisfaction (*r* = 0.47, *p* < 0.001). Analyzing the specific dimensions of the construct, emerged how only being-away was positively correlated with physical and psychological well-being (*r* = −0.33, *p* = 0.022) and work engagement (*r* = 0.36, *p* = 0.014). However, even if it was not statistically significant, the correlation between physical and psychological well-being and coherence was −0.26 (*p* = 0.084). Fascination and coherence positively correlated with work engagement and job satisfaction, highlighting the role of restorativeness dimensions also on the organizational outcomes.

## Discussion

This study describes the process and the results of a research-intervention project aimed at designing a workplace, using a participatory design process and following some guidelines coming from environmental psychology research results and the biophilic design approach. The research-intervention was aimed at designing the new site of an Italian non-profit organization, and the process was carried on in the old site. Employees and managers have been involved in this process, together with an interdisciplinary team of experts, including architects, technicians of the organization, and some environmental psychology researchers. At the end, based on the shared drawings and shared ideas, the new site has been designed and the project has been realized. Employees have been also involved in an assessment procedure, aimed at evaluating individual perceptions of the physical environment, including some important aspects in line with biophilic design perspective, as well as some measures of psychological well-being. These last variables allow to empirically verify the connection with physical aspects of the workplace—actually their individual perceptions—and psychological well-being. At the moment, few research has been designed to give empirical evidence of what is deeply explored in the literature. Furthermore, still less are research on this topic which joined participatory design methodologies. At the end of the process, the new site was implemented, and employees moved into the new workplace. The assessment was carried considering both in the old site (Time 1) and in the new site (T2) for all the variables concerning the physical environment perception, to verify the improvement of some aspects connected with biophilic design. Physical and psychological well-being, measured with General Health Questionnaire (Goldberg and Williams, 2000), a standardized scale which is often used to measure psychological functioning, was also assessed twice, to explore whether an improvement in Physical and psychological well-being could be found in moving through the two workplaces. At the end, psychological well-being directly connected with work experience, such as work engagement and job satisfaction, was evaluating 2 months after the use of the new site.

The new site has been designed following some important characteristics according to biophilic design guideline. The goal of biophilic design is not simple, and well-recognized guidelines for a concrete implementation are not easy to find. Kellert (2018) suggested 72 design attributes grouped in six elements: environmental features, natural shapes and forms, natural patterns and processes, light and space, place-based relationships and evolved human-nature relationships. Browning and Ryan (2020) propose 15 patterns of biophilic design which they group in three categories: nature in the space, natural analogs and nature of the space. Bolten and Barbiero (2020), propose a synthesis which includes light, protection and control, air, views, greenery, curiosity, and materials, finishing and colors. Based on these approaches, our research-intervention highlighted the importance of some physical elements in the design of the new workplace: light, considering both the presence and quality of natural light and the personal control on light, air quality, the presence of view connected with nature, the acoustic comfort, and the quality of destress areas. These five aspects were considered, among others, in the design process, and at the same time the survey assessed the perceived quality and satisfaction on these five aspects, both for the old site (T1) and the new one (T2). Furthermore, perceived restorativeness of the workplace has been studied, considering three important dimensions applicable to the workplace experience: being-away, fascination and coherence, together with the overall perceived restorativeness.

Survey results showed that perceived quality and satisfaction for the physical environment features significantly improved in all the five considered aspects, comparing the new site with the old one. The overall perceived restorativeness also improved, mainly due to a significant improvement in coherence, but also to a small improvement in fascination. Coherence and fascination are two important dimensions to be considered, and a physical environment which enhance a perfect balance between coherence and fascination, high legibility and curiosity, can reduce mental fatigue and help to avoid other sources of stress in workers. Stress in the workplace can occur when there is an imbalance between physical setting demands and human resources, because individuals cannot cope with demands in the proper way. In fact, physical and mental resources put in jeopardy by stress due to the workplace physical characteristics can lead to a lack of well-being in the work context.

Results also highlighted the relationship between perceived quality of the five aspects of work environment and the perceived restorativeness. This correlation has been found for overall perceived restorativeness and all the five physical characteristics in the second assessment, and for all aspects except one in the first assessment. However, it is important to note that the low variability of measures at T1 could have lowered the correlations between these variables. This relationship between restorativeness and physical aspects of the workplace, as light, air, acoustic comfort, view of natural element and the quality of destress areas, as far as we know, has not been explored, and this is one of the first study that empirically describes this result.

An important result consists in the significant relationship between the perceived quality and satisfaction for the five physical aspects or the workplace, and the well-being outcomes, such as psychophysical well-being, work engagement and job satisfaction. As shown by the correlation analysis, the improvements in the perceived quality and satisfaction in four of the five physical aspects, were strongly linked with job satisfaction and slightly less with work engagement.

Weaker seemed the relations with physical and psychological well-being, which was found related only to the view connected with nature. Probably this weaker relation and the absence of the effect of the other physical aspects could be explained considering the pandemic context which could have influenced employees' perceptions especially on personal health. However, it also highlighted the strength of the view connected with nature which, also in pandemic situation seemed to have the possibility to positively affect physical and psychological health.

The present research also confirmed the positive relationship between restorativeness dimensions and organizational outcomes. The positive effect of restorativeness and job satisfaction and work engagement have been already documented (Bellini et al., 2015a,b, 2019). Organizational outcomes are also affected by the quality of the physical aspects of workplace (Leder et al., 2016). A possible explanation, considering the psychological mechanisms behind this relationship, is the mediation role of restorativeness in the relationship between physical characteristics of the workplace and well-being at work.

Being-away is the only restorativeness dimension that is significantly linked to the physical and psychological well-being. Maybe, the other aspects of restorativeness are less directly connected with general psychological and physical health, whereas the possibility of feeling away by the everyday routine is an actually important aspect.

Furthermore, the results of the present research-intervention highlighted how the use of the participatory techniques added a great value to the biophilic design application process. At the same time, it grew employees' awareness about the biophilic design potential in increasing their well-being at the workplace. Furthermore, participatory techniques allowed to design environments more effectively, thanks to the knowledge, needs and wishes of employees developed in the previous site. Moreover, this, to our knowledge, is the first case in which the effectiveness of biophilic design realized through participatory techniques has been empirically tested.

Beyond the discussed results, that try to shine a small light on this topic, using a research-intervention design, this study presents some limitations, which must be highlighted. First of all, the study was conducted during the pandemic period due to the COVID-19, and this situation could have influenced the results. Workers spent much more time at home, working remotely, and this could have affected their evaluation of the workplace, in which they spent less time than usual.

Another limitation regards the small sample size. The number of the workers of this organization was not so large, and, mainly during the first survey, a response rate of 71% (considering the workers that completed the survey) make this initial number not so high. The number of workers involved in the second assessment was larger, and also the response rate increased (89%), but only 26 workers completed the first and the second assessment, allowing a comparison, that is the 67% of the workers which answered to the first survey. This limitation should be considered in the double sense: whereas some significant results in line with expected theoretical results have been found, it is not possible to establish whether other not-significant results could be due to the small sample size.

A limited internal validity is also connected with the confusion between the qualities of the physical environment of the workplace, which maybe actually improved in the new site, explaining the increasing of workers' satisfaction, and the novelty effect. It is impossible to establish whether a new site, planned with different criteria, not in line with biophilic design principles, would be positively evaluated in a similar way. It should have been interesting to control for this novelty effect, assessing the same variables in a different group of workers, involved only in a site change, with no attention paid to the biophilic characteristics of the new workplace. In the future, this possibility should be considered from the beginning. Moreover, other important variables generally used in environmental psychology could have been considered. One of this is place attachment (Scrima, 2015): attachment to the workplace maybe act as a moderator, buffering the positive effect of the new site.

A future direction of this specific research intervention is to do a third wave assessment, to verify the persistence of the increased evaluation of the quality of the physical environment of the new site, after a longer period of work in the workplace. This third assessment has already been planned, for the next months, and it will be really important, also considering the more time spent working in the office instead of remotely, due to the pandemic restrictions reduction.

## Data Availability Statement

The raw data supporting the conclusions of this article will be made available by the authors, without undue reservation.

## Ethics Statement

Ethical review and approval was not required for the study on human participants in accordance with the local legislation and institutional requirements. The patients/participants provided their written informed consent to participate in this study.

## Author Contributions

MP and MB organized the database and performed the statistical analysis. All authors contributed to conception and design of the study and contributed to write the first draft of the manuscript, and on the revision, read, and approved the submitted version.

## Conflict of Interest

The authors declare that the research was conducted in the absence of any commercial or financial relationships that could be construed as a potential conflict of interest.

## Publisher's Note

All claims expressed in this article are solely those of the authors and do not necessarily represent those of their affiliated organizations, or those of the publisher, the editors and the reviewers. Any product that may be evaluated in this article, or claim that may be made by its manufacturer, is not guaranteed or endorsed by the publisher.
